# Is treated HIV infection associated with knee cartilage degeneration and structural changes? A longitudinal study using data from the osteoarthritis initiative

**DOI:** 10.1186/s12891-019-2573-5

**Published:** 2019-05-04

**Authors:** Yao Liu, Sarah C. Foreman, Gabby B. Joseph, Jan Neumann, Phyllis C. Tien, Xiaoming Li, Nancy E. Lane, Michael C. Nevitt, Charles E. McCulloch, Thomas M. Link

**Affiliations:** 1 Musculoskeletal Quantitative Imaging Research Group, Department of Radiology and Biomedical Imaging, University of California San Francisco, 185 Berry St, Suite 350, San Francisco, CA 94107 USA; 2 Department of Radiology, Tongji Hospital, Tongji Medical College, Huazhong University of Science and Technology, Wuhan, China; 3 Division of Infectious Diseases, Department of Medicine, University of California, San Francisco, CA USA; 4 Veterans Affairs Medical Center, San Francisco, CA USA; 5 Department of Internal Medicine, UC Davis Medical Center, Sacramento, CA USA; 6 Department of Epidemiology and Biostatistics, University of California San Francisco, San Francisco, CA USA

**Keywords:** HIV, Antiretroviral therapy, Knee, Cartilage, Osteoarthritis

## Abstract

**Background:**

Metabolic disorders presenting in HIV-infected patients on antiretroviral therapy (ART) may increase the risk of osteoarthritis. However, structural changes of the knee in HIV infected subjects are understudied. The aim of this study is to investigate knee cartilage degeneration and knee structural changes over 8 years in subjects with and without HIV infection determined based on the use of ART.

**Methods:**

We studied 10 participants from the Osteoarthritis Initiative who received ART at baseline and 20 controls without ART, frequency matched for age, sex, race, baseline body mass index (BMI) and Kellgren & Lawrence grade. Knee abnormalities were assessed using the whole-organ magnetic resonance imaging score (WORMS) and cartilage T2 including laminar and texture analyses were analyzed using a multislice-multiecho spin-echo sequence. Signal abnormalities of the infrapatellar fat pad (IPFP) and suprapatellar fat pad (SPFP) were assessed separately using a semi-quantitative scoring system. Linear regression models were used in the cross-sectional analysis to compare the differences between ART/HIV subjects and controls in T2 (regular and laminar T2 values, texture parameters) and morphologic parameters (subscores of WORMS, scores for signal alterations of IPFP and SPFP). Mixed effects models were used in the longitudinal analysis to compare the rate of change in T2 and morphological parameters between groups over 8 years.

**Results:**

At baseline, individuals on ART had significantly greater size of IPFP signal abnormalities (*P* = 0.008), higher signal intensities of SPFP (*P* = 0.015), higher effusion scores (*P* = 0.009), and lower subchondral cysts sum scores (*P* = 0.003) compared to the controls. No significant differences were found between the groups in T2-based cartilage parameters and WORMS scores for cartilage, meniscus, bone marrow edema patterns and ligaments (*P* > 0.05). Longitudinally, the HIV cohort had significantly higher global knee T2 entropy values (*P* = 0.047), more severe effusion (*P* = 0.001) but less severe subchondral cysts (*P* = 0.002) on average over 8 years.

**Conclusions:**

Knees of individuals with HIV on ART had a more heterogeneous cartilage matrix, more severe synovitis and abnormalities of the IPFP and SPFP, which may increase the risk of incident knee osteoarthritis.

**Electronic supplementary material:**

The online version of this article (10.1186/s12891-019-2573-5) contains supplementary material, which is available to authorized users.

## Background

Since the introduction and constant optimization of antiretroviral therapy (ART), the life expectancy of people living with HIV (PLWH) has increased significantly over the past decades and is now similar to or approaching that of an HIV-negative person [1]. However, the direct or indirect consequences of immunodeficiency, chronic inflammatory status and cumulative toxic effects of ART, aging patients with HIV infection are reported to have increased risk for “non-AIDS” comorbidities such as cardiovascular diseases, neurocognitive diseases, liver and kidney diseases, metabolic syndrome (MetS), cancers and rheumatic diseases [1–4]. With the increasing life expectancy of PLWH, the impact of these “non-AIDS” comorbidities are gaining clinical significance [5].

Osteoarthritis (OA) is one of the most common rheumatological disorders, which affects 240 million people globally and 27 million people in the USA [6, 7]. Although aging remains the most important risk factor for OA, MetS, which is a combination of hypertension, dyslipidemia, diabetes (or insulin resistance) and abdominal obesity, may also play an essential role in the occurrence and development of OA [8]. MetS also affects up to half of HIV-infected patients receiving ART [9]. While studies have reported an increased prevalence and severity of radiographic hand osteoarthritis in patients with HIV-1 infection, in particular associated with MetS, the exact pathophysiology of OA in PLWH is not well understood [10, 11]. Studies have reported more heterogeneous cartilage T2 relaxation times in diabetics, indicating increased articular cartilage degeneration [12, 13]. The detrimental impact of dyslipidemia, obesity and MetS-associated chronic low-grade inflammation on cartilage metabolism have also been demonstrated [8, 14, 15].

A recent cross-sectional study [16] suggested that tibial cartilage volume of HIV-infected patients was reduced and associated with increased central fat accumulation, which may potentially influence the subsequent development of knee OA. However, there is limited knowledge about the association of HIV/ART with knee OA. In particular, little is known how HIV and ART affect the composition of knee cartilage and how they affect the severity and progression of knee OA.

Torshizy et al. [17] and Petscavage et al. [18] described MR imaging signal alterations of the infrapatellar fat pad (IPFP) and the suprapatellar fat pad (SPFP) in PLWH with non-specific knee pain, that was characterized by global homogeneous increase in signal throughout IPFP or SPFP on fluid sensitive sequences. The IPFP, also known as Hoffa’s fat pad, not only plays a role in absorbing mechanical force generated by joint movement but affects the occurrence and development of knee OA by producing and releasing important inflammatory mediators [19]. Several studies [20–24] have reported that signal intensity alterations of the IPFP and the SPFP are important imaging biomarkers for knee OA. In the setting of HIV, however, it is not known whether and how signal alteration within the IPFP and SPFP change over time and whether it increases the risk of OA in HIV-infected patients.

Thus, the purpose of our study was to evaluate knee joint health in individuals with HIV treated with ART using quantitative and structural MRI-based parameters and compare these with matched controls using a longitudinal study design.

## Methods

### Database and subjects

This study utilized data from the Osteoarthritis Initiative cohort (OAI, https://oai.nih.gov), a longitudinal, multi-center cohort study which recruited 4796 individuals and is sponsored by the US National Institutes of Health (NIH). The aim of the OAI was to develop a large database to study the natural progression, risk factors and predictors of knee OA. The study protocol, amendments, and informed consent documentation were reviewed and approved by all local institutional review boards. Information about specific OAI datasets that we used are described in Additional file 1.

Subjects with HIV infection were identified based on the use of typical HIV ART combinations at baseline as recorded in the baseline medication inventory, a database of prescription medications taken by participants in the past 30 days (Additional file 2). Inclusion criteria required subjects in the HIV cohort to be on ART regime for at least one year and for control subjects to not take any type of antiretroviral medications during the follow-up period. HIV subjects were frequency matched with control subjects 1:2 for age, sex, race, baseline body mass index (BMI) and Kellgren-Lawrence (K&L) grades. Categories for the matching were defined by combination of: sex (male or female), K&L grade (0, 1, 2, 3, 4), age (5 year intervals), BMI (2.5 kg/m^2^ intervals) and race (White or Caucasian, Black or African American). Individuals with a history of rheumatoid arthritis were excluded.

### MR imaging protocol

MR images were obtained at four different clinical sites of the OAI with cross calibrated 3.0-T scanners (Magnetom Trio, Siemens, Erlangen, Germany) using identical quadrature transmit-receive coils (USA Instruments, Aurora, Ohio). To semi-quantitatively assess structural abnormalities of bilateral knees three sequences were used: (a) coronal 2D intermediate-weighted (IW) turbo spin-echo (TSE) sequence (repetition time [TR]/echo time [TE] = 3700/29 ms), (b) sagittal 3D dual-echo steady-state (DESS) sequence with water excitation and coronal and axial reformations (TR/TE = 16.3/4.7 ms, flip angle = 25°) and (c) sagittal 2D IW fat-suppressed TSE sequence (TR/TE = 3200/30 ms).

Quantitative measurements based on T2 relaxation time were obtained of the right knee using a sagittal 2D multislice multiecho (MSME) spin-echo (SE) sequence (TR = 2700 ms, TEs = 10, 20, 30, 40, 50, 60 and 70 ms, field of view = 12 cm, slice thickness = 3 mm with 0.5 mm gap, in-plane spatial resolution = 0.31 × 0.45 mm^2^). This sequence was only performed at the right knee. Additional information about the above sequences are available in the OAI MR protocol [25].

### MR image analysis

#### Semi-quantitative morphological analysis

All available images of both left and right knees at baseline and annual follow-up timepoints (12-months, 24-months, 36-months, 48-months, 72-months, 96-months; the numbers of participants with available images at each time point are summarized in Additional file 3) of HIV subjects and controls were assessed by one radiologist (Y.L. 4 years of experience) blinded to subject characteristics and under supervision of a board certified musculoskeletal radiologist (T.M.L. 24 years of experience). The modified WORMS [26] was used for semi-quantitative analysis of OA-related abnormalities. The following parameters were evaluated separately: meniscal lesions were graded from 0 to 5 in each of 6 regions (medial/lateral and anterior/body/posterior); cartilage defects were graded from 0 to 6, bone marrow edema pattern (BMEP) as well as subarticular cysts were scored from 0 to 3 in each of 6 regions (patella, trochlea, medial/lateral femur, and medial/lateral tibia). Other lesions including ligamentous abnormalities and joint effusion were also scored. Moreover, we calculated sum scores for each imaging parameter individually over all sub-regions of each knee.

#### T2 relaxation time measurements

The software used for the T2 analysis was developed at our institution and is a spline-based algorithm written in MATLAB (the Mathworks, Natick, Massachusetts), that has been described previously [13]. The cartilage of five compartments (patella, lateral femur, medial femur, lateral tibia, medial tibia) was semi-automatically segmented. The trochlear region was excluded from the analysis due to flow-artifacts caused by the popliteal artery. Mean T2 values of the baseline and available follow-up timepoints were computed separately for each compartment and globally (average of all compartments combined) of the segmented regions of interest.

#### Laminar and GLCM texture analysis

Laminar and gray level co-occurrence matrix (GLCM) texture analyses were performed to characterize the spatial distribution of cartilage T2 values for each compartment (patella, lateral femur, medial femur, lateral tibia, medial tibia) and the whole knee joint (the mean was calculated across all compartments). Laminar analysis can reveal early laminar disruption within cartilage when mean T2 does not yet show changes of cartilage composition [12, 13]. In the laminar analysis, the cartilage in each compartment was split into two layers with approximately the same thickness, a deep layer adjacent to the bone-cartilage interface (referred to as the bone layer) and an articular superficial layer (referred to as the articular layer). For GLCM texture analysis, contrast, variance, and entropy were computed to measure the extent of heterogeneity of T2 values within the cartilage matrix, which has been used to detect compositional changes of cartilage before changes of regular T2 values is present [13, 27]. Contrast characterizes the differences of grey levels between each pixel and its neighboring pixels with elevated T2 contrast demonstrating higher differences and more heterogeneity. Variance assesses the distribution of pixels about the mean, indicating how many pixels vary from the average compartment grey level. Entropy measures the disorder in an image, with high entropy values suggesting less uniform distributions of probabilities of T2 value co-occurrences.

#### IPFP and SPFP signal abnormalities analysis

Using sagittal 2D IW fat-suppressed TSE sequences, two radiologists (T.M.L. and Y.L.) assessed the size and highest signal intensity of IPFP signal abnormalities and the highest signal intensity of SPFP signal abnormalities (due to the small volume of the SPFP, the size of signal abnormalities was not assessed). The size of IPFP signal abnormalities was graded as follows: grade 0 = none; grade 1 ≤ 33% of the region; grade 2 = 34–66% of the region; grade 3 ≥ 66% of the region; while the highest signal intensity was characterized as follows: grade 0 = none; grade 1 = mild (lower than cartilage); grade 2 = moderate (equal to or higher than cartilage but lower than fluid); grade 3 = severe (equal to fluid).

### Intra- / inter-reader reproducibility

Reproducibility results of the WORMS grading and the knee cartilage T2 relaxation time measurements have been described and validated by our group in multiple previous studies [12, 13, 26, 28]. For WORMS readings, the intra-class correlation coefficients (ICCs) for intrareader agreement ranged from 0.85 to 0.98 for meniscus and 0.84 to 0.97 for cartilage, and ICCs for inter-reader agreement ranged from 0.83 to 0.95 for meniscus and 0.80 to 0.92 for cartilage. For reproducibility of cartilage T2 measurements, coefficients of variation (CV) were calculated on a percentage basis as the root mean square average, with CVs ranging from 1.66 to 1.76% for intrareader agreement and 1.12 to 2.06% for inter-reader agreement.

Reproducibility for IPFP and SPFP gradings were assessed in 10 randomly selected subjects. Each score of the gradings was graded twice by two radiologists (T.M.L. and Y.L.) on two separate occasions with a separation of 4 weeks in between those two readings. Observed agreement and Weighted Cohen’s Kappa values were calculated to compare each score separately. Intra-reader observed agreement/kappa values were 95%/0.87 and 88%/0.64 for the size of IPFP signal abnormalities, 95%/0.86 and 85%/0.55 for the highest signal intensity of IPFP signal abnormalities, 95%/0.77 and 90%/0.00 (the observed agreement and chance agreement were equal) for highest signal intensity of SPFP signal abnormalities. Inter-reader observed agreement/kappa values were 98%/0.94 for the size of IPFP signal abnormalities, 93%/0.80 for the highest signal intensity of IPFP signal abnormalities and 95%/0.77 for the highest signal intensity of signal abnormalities within SPFP.

### Statistical analysis

Statistical analyses were performed with STATA version 14 software (StataCorp LP, College Station, TX), using a two-sided, 0.05 level of significance. The differences in baseline subject characteristics between groups were assessed using Student’s independent t-tests (continuous variables) and chi-square tests (categorical variables). For the cross-sectional analysis, linear regression models were used to assess the differences between ART/HIV subjects and controls in T2 (regular and laminar T2 values, GLCM texture parameters) and morphologic parameters (subscores of WORMS, scores for signal alterations of IPFP and SPFP). Mixed effects models were used in the longitudinal analysis to compare the rate of change in T2 and morphological parameters between groups over 8 years by testing for an interaction between time and HIV group. For the differences in the rates of change over time, we first tested for non-linearity by including an interaction between time [quadratic] and HIV group. If the quadratic time term was significant (*P* < 0.05), then we used a quadratic, non-linear model with an interaction between quadratic time and HIV group. If quadratic time term was not significant then we used time [linear] interaction with HIV group in the model. If there were no significant interactions of HIV group with time (*P* > 0.05) we tested whether there were significant differences in T2 and morphological parameters averaged over all timepoints. All analyses were adjusted for baseline age, sex, race, baseline BMI and K&L grades.

Based on results from previous studies [12, 13], we considered T2-based knee cartilage parameters (cartilage T2 values, laminar T2 values and GLCM texture parameters) as primary outcome measures, while other morphological parameters (cartilage damage, meniscus lesions, BMEPs, subchondral cysts, effusion and ligamentous abnormalities) and signal abnormalities of IPFP and SPFP were considered exploratory outcome parameters.

## Results

Our sample consisted of 10 subjects in the ART/HIV group and 20 subjects in the control group (30% female, mean baseline age 52.1 ± 6.2 years, mean baseline BMI 27.6 ± 3.7 kg/m^2^). As expected, based on frequency matching, no significant differences were noted for age, sex, race, and baseline BMI and K&L grades between subjects with and without HIV infection (Table 1).

**Table 1:** Subject characteristics at baseline

	All subjects (*n* = 30)	HIV group (*n* = 10)	Control group (*n* = 20)	*P* value^a^
Age, mean ± SD years	52.1 ± 6.2	52.2 ± 6.9	52.0 ± 6.0	0.940
Females [n (%)]/Males [n (%)]	9 (30%) / 21 (70%)	3 (30%) / 7 (70%)	6 (30%) / 14 (70%)	1.000
BMI, mean ± SD kg/m^2^	27.6 ± 3.7	27.4 ± 3.6	27.7 ± 3.8	0.831
Knee Kellgren-Lawrence scores				1.000
Grade 0 [n (%)]	27 (45%)	9 (45%)	18 (45%)	
Grade 1 [n (%)]	12 (20%)	4 (20%)	8 (20%)	
Grade 2 [n (%)]	15 (25%)	5 (25%)	10 (25%)	
Grade 3 [n (%)]	6 (10%)	2 (10%)	4 (10%)	
Racial composition				1.000
White or Caucasian [n (%)]	12 (40%)	4 (40%)	8 (40%)	
Black or African American [n (%)]	18 (60%)	6 (60%)	12 (60%)	

Continuous data were expressed as mean ± SD. Categorical data were presented in numbers of participants with percentage in parentheses. ^**a**^ *P*-values were calculated using either Pearson’s χ^2^-test or independent t-test as appropriate

### Primary outcome parameters

#### Cartilage matrix composition

At baseline, global overall T2 values, global T2 values of bone and articular layer as well as all global T2 texture parameters (contrast, variance, and entropy) were slightly higher in the HIV group compared to the control group, but no significant differences were observed (Table 2).

**Table 2:** Results of baseline analyses for primary and exploratory outcome parameters between groups

Parameters	Control group (*n* = 20)	HIV group (*n* = 10)	Effect size (95% CI)^b^	*P* value
	Adjusted means^a^	Adjusted means^a^		
Primary outcome parameters				
Regular T2 values				
Global knee T2	32.71 ± 0.43	32.90 ± 0.60	0.19 (−1.35, 1.73)	0.801
Laminar T2 values				
Global bone layer T2	29.92 ± 0.37	30.14 ± 0.52	0.22 (− 1.10, 1.54)	0.737
Global articular layer T2	34.85 ± 0.50	36.03 ± 0.71	1.17 (−0.62, 2.97)	0.188
Texture parameters				
Global knee contrast	304.98 ± 16.45	307.59 ± 23.27	2.61 (− 56.39, 61.61)	0.928
Global knee variance	223.60 ± 11.76	224.22 ± 16.64	0.61 (−41.57, 42.79)	0.976
Global knee entropy	6.29 ± 0.04	6.40 ± 0.05	0.11 (− 0.03, 0.25)	0.124
Exploratory outcome parameters				
WORMS scores				
Cartilage sum score	4.15 ± 0.49	3.15 ± 0.69	−1.00 (− 2.65, 0.66)	0.238
Meniscus sum score	2.58 ± 0.44	1.68 ± 0.62	−0.90 (− 2.40, 0.60)	0.241
BMEPs sum score	2.60 ± 0.38	1.86 ± 0.53	−0.74 (− 2.02, 0.54)	0.260
Subchondral cysts sum score	2.15 ± 0.30	0.65 ± 0.42	−1.51 (−2.51, −0.50)	**0.003**
Effusion score	0.05 ± 0.04	0.25 ± 0.06	0.20 (0.05, 0.35)	**0.009**
Ligament sum score	0.45 ± 0.15	0.31 ± 0.21	−0.14 (−0.66, 0.37)	0.590
IPFP and SPFP signal abnormalities				
IPFP signal abnormalities (size)	0.78 ± 0.13	1.39 ± 0.19	0.62 (0.16, 1.07)	**0.008**
IPFP signal abnormalities (SI)	1.30 ± 0.17	1.55 ± 0.24	0.24 (−0.34, 0.83)	0.409
SPFP signal abnormalities (SI)	1.35 ± 0.11	1.80 ± 0.16	0.45 (0.09, 0.81)	**0.015**

^a^ adjusted means were expressed as mean ± SD. ^**b**^ effect size for difference in each parameter between groups, with (95% confidence intervals, CI) provided. Adjusted means and effect size were corrected for age, sex, race and baseline BMI and K&L grades. *P* values < 0.05 were in bold, indicating significant difference between two groups in corresponding score. *BMEP* bone marrow edema pattern, *IPFP* infrapatellar fat pad, *SPFP* suprapatellar fat pad, *SI* signal intensity

**Fig. 1:**
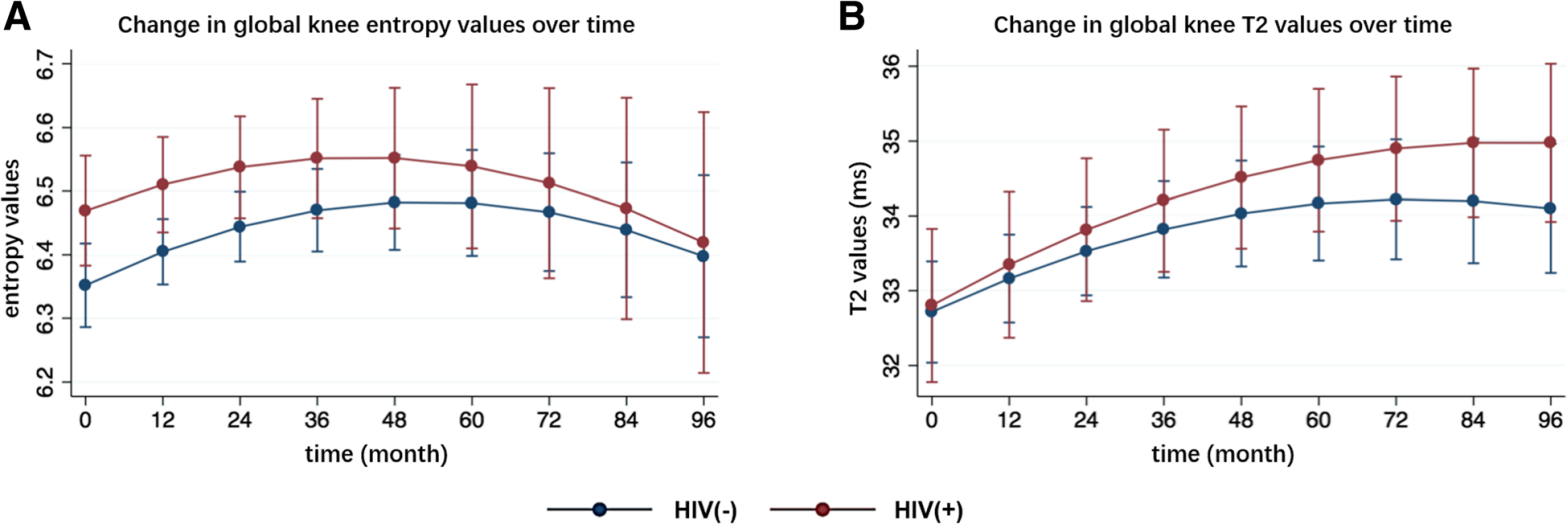
Changes in global entropy and global T2 values during the 96-month follow-up. **a** Changes in global entropy values over 96 months. **b** Changes in global knee T2 values over 96 months. Data were given as adjusted means, corrected for age, sex, race and baseline BMI and K&L grades. Error bars represented standard errors. *P*-values in bold refer to significant differences between groups on average over 8 years, respectively: global T2 entropy (*P* = 0.047), global T2 (*P* = 0.809)

The interaction between time and the HIV group was significant for the texture parameters global knee contrast and variance (Table 3). On average over all timepoints, global knee entropy values were significantly higher in the HIV group compared to the control group (*P* = 0.047, Fig. 1a). Global T2 values (*P* = 0.809, Fig. 1b), global T2 values of bone layer (*P* = 0.669) and articular layer (*P* = 0.180) were slightly higher on average over all timepoints in the HIV group but not significantly (*p* > 0.05).

**Table 3:** Results of longitudinal analyses for primary and exploratory outcome parameters between groups

Parameters	The rates of change over 8 years		Differences on average over 8 years	
	Effect size (95% CI)^a^	*P*_i_ value	Effect size (95% CI)^b^	*P*_a_ value
Primary outcome parameters				
Regular T2 values				
T2 global*	0.005 (−0.011, 0.022)	0.523	0.13 (−0.90, 1.15)	0.809
Laminar T2 values				
Global bone layer T2	0.006 (−0.009, 0.022)	0.411	0.20 (−0.72, 1.12)	0.669
Global articular layer T2	0.005 (−0.014, 0.024)	0.620	0.89 (−0.41, 2.19)	0.180
Texture parameters				
Global knee contrast*	−0.072 (−0.108, − 0.035)	**0.000**		
Global knee variance*	−0.031 (− 0.050, − 0.013)	**0.001**		
Global knee entropy*	−0.001 (− 0.004, 0.002)	0.460	0.09 (0.00, 0.19)	**0.047**
Exploratory outcome parameters				
WORMS scores				
Cartilage sum score	0.012 (−0.008, 0.032)	0.248	−1.04 (−2.61, 0.53)	0.194
Meniscus sum score	0.004 (−0.008, 0.015)	0.514	−0.79 (− 2.31, 0.74)	0.313
BMEPs sum score	0.004 (−0.006, 0.015)	0.437	−0.89 (−2.21, 0.43)	0.188
Subchondral cysts sum score	0.001 (−0.009, 0.012)	0.788	−1.51 (−2.47, − 0.55)	**0.002**
Effusion score	0.000 (−0.003, 0.003)	0.865	0.24 (0.10, 0.38)	**0.001**
Ligament sum score*	−0.001 (− 0.007, 0.004)	0.610	− 0.16 (− 0.65, 0.34)	0.534
IPFP and SPFP signal abnormalities				
IPFP signal abnormalities (size)	−0.007 (− 0.011, − 0.003)	**0.001**		
IPFP signal abnormalities (SI)	−0.006 (− 0.011, − 0.002)	**0.010**		
SPFP signal abnormalities (SI)*	−0.004 (− 0.007, − 0.000)	**0.030**		

*Parameters that had a non-linear change over 8 years. ^a^Effect size for the rates of change over 8 years between groups, with (95% confidence intervals, CI) provided. ^b^Effect size for the differences on average over 8 years between groups, with (95% confidence intervals) provided. Effect size were corrected for age, sex, race and baseline BMI and K&L grades. *P*_i_ values < 0.05 were in bold, indicating significant interactions with time. *P*_a_ values < 0.05 were in bold, indicating significant differences on average over 8 years. *BMEP* bone marrow edema pattern, *IPFP* infrapatellar fat pad, *SPFP* suprapatellar fat pad, *SI* signal intensity

### Exploratory outcome parameters

#### Knee morphological changes

At baseline, the HIV group had significantly lower subchondral cysts WORMS sum scores and significantly higher effusion WORMS scores (*P* = 0.003 and *P* = 0.009 respectively, Table 2). No differences were observed between groups in WORMS sum scores for cartilage, meniscus, BMEPs and ligaments.

**Fig. 2:**
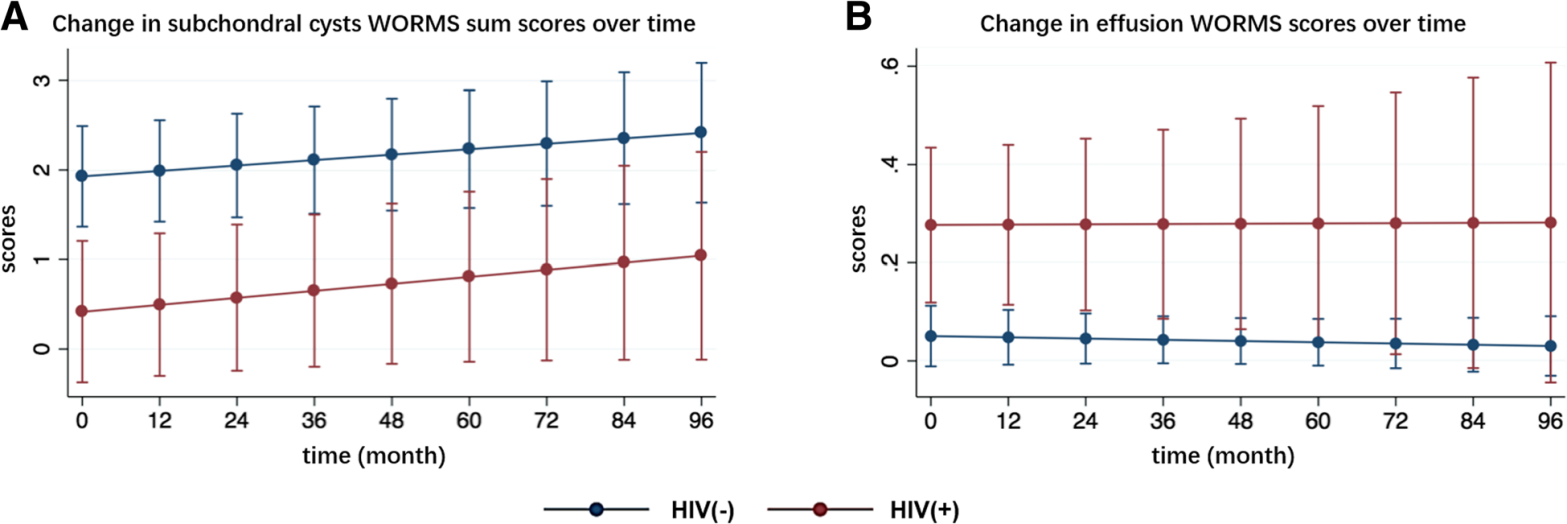
Changes in subchondral cysts WORMS sum scores and effusion WORMS scores during the 96-month follow-up. **a** Changes in subchondral cysts WORMS sum scores over 96 months. **b** Changes in effusion WORMS scores over 96 months. Data were given as adjusted means (scores), corrected adjusted for age, sex, race and baseline BMI and K&L grades. Error bars represented standard errors. *P*-values in bold refer to significant differences between groups on average over 8 years, respectively: subchondral cysts sum scores (*P* = 0.002) and effusion scores (*P* = 0.001)

The interaction between time and the HIV group was not significant for all morphological parameters assessed based on WORMS (Table 3). On average over all timepoints, subchondral cysts sum scores were found to be significantly lower in PLWH (*P* = 0.002, Fig. 2a), while effusion scores were significantly higher compared to controls (*P* = 0.001, Fig. 2b). The WORMS sum scores for cartilage, meniscus, BMEPs and ligaments were not significantly different on average over all time points between groups.

#### IPFP and SPFP signal abnormalities

**Fig. 3:**
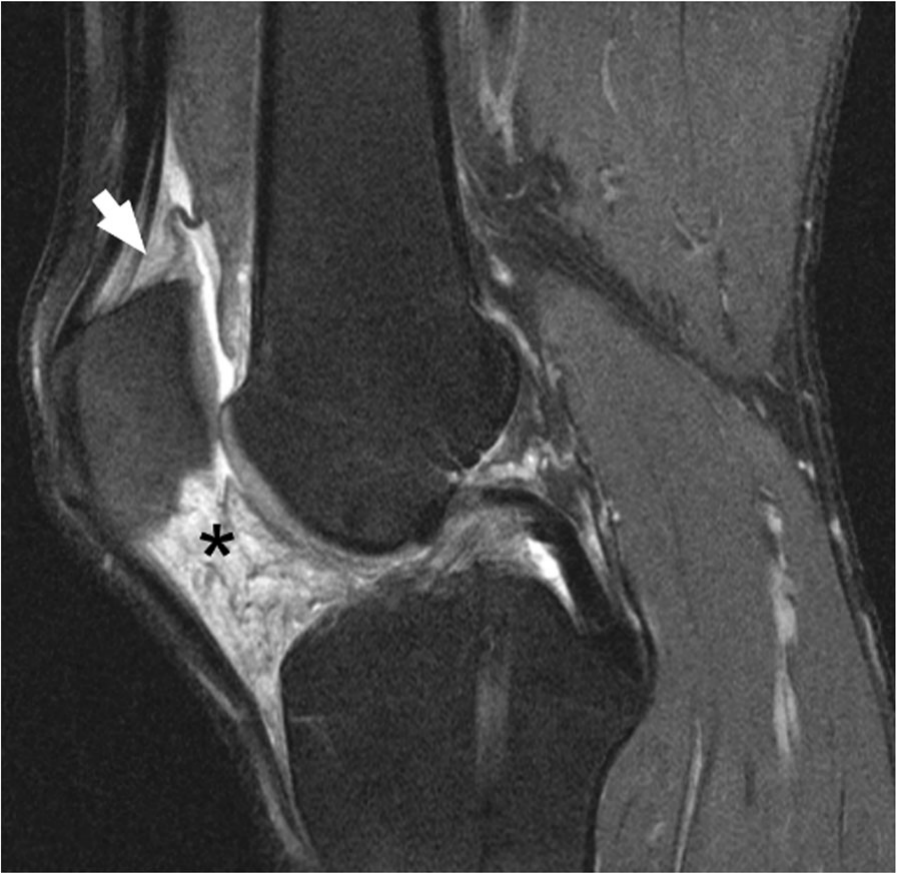
46-year-old HIV-infected man on ART with diffuse increased signal intensity within infrapatellar fat pad. Sagittal intermediate-weighted fat-suppressed image (TR/TE = 3200/30 ms, FOV = 16 cm, slice thickness = 3 mm) of the left knee in a 46-year-old HIV-infected male on ART presented diffuse increased signal intensity within infrapatellar fat pad (*), and to a lesser extent within suprapatellar fat pad (arrow)

The baseline scores for the size of IPFP signal abnormalities and the signal intensity scores of the SPFP were significantly higher in the HIV group (*P* = 0.008 and *P* = 0.015 respectively, Table 2). The signal intensity scores of the IPFP were also higher in HIV cohort, however, this was not statistically significant (*P* = 0.409). At baseline, two out of the ten HIV subjects had a diffuse homogeneously increased signal intensity throughout the whole IPFP on fluid sensitive sequences (Fig. 3), which was not observed in any of the 20 HIV negative subjects.

The interaction between time and the HIV group was significant for all fat pad parameters (Table 3), therefore significant differences averaged over all timepoints were not tested.

## Discussion

This is the first longitudinal 3 T MRI study to evaluate quantitatively and semi-quantitatively knee degenerative changes and signal alteration of the IPFP and SPFP in people living with HIV (PLWH) treated with ART. We found that PLWH had higher global knee entropy T2 values averaged over all timepoints in the longitudinal analysis, indicating a more heterogeneous and disordered cartilage matrix composition. At baseline, we found that the HIV group had significantly greater size of IPFP signal abnormalities and higher signal intensity of the SPFP. Moreover, PLWH had significantly higher joint effusion scores at baseline and on average over all timepoints compared to the control group.

Cartilage damage is the hallmark of knee OA, and cartilage matrix degeneration is an essential event that determines the irreversible progression of knee OA [29]. To date, only one study reported that total body fat and android fat were inversely associated with knee tibia cartilage volume in PLWH [16]. In our study, PLWH showed a more heterogeneous and disordered cartilage matrix composition in the longitudinal analysis of cartilage GLCM texture parameters. Interestingly, we did not find any significant differences in WORMS cartilage scores, indicating that findings in HIV infection may predominantly affect the knee cartilage matrix. However due to small sample size we may not have had enough power to detect significant differences. The exact mechanism of cartilage degeneration in PLWH remains to be elucidated. In the setting of HIV-related joint disease there are at least two aspects worth considering: First, the HIV-associated sarcopenia may reduce the strength of muscle around the knee joint and affect the normal knee joint biomechanics [30], which may promote cartilage degeneration. The fatty infiltration in the muscles surrounding the knee joint may also be involved in the alteration of muscle strength [4, 31]. Secondly, multiple metabolic abnormalities associated with HIV, medications, and chronic inflammation have been reported to have deleterious effects on cartilage metabolism, including hypertension, hyperglycemia, lipodystrophy among others [2, 4, 8, 13–15]. For example, subchondral ischemia resulting from hypertension may compromise the nutrient exchange of cartilage, and hyperglycemia may induce cartilage matrix stiffness, subchondral bone destruction and chondrocyte dysfunction [14]. To date, little is known about whether HIV/ART can directly affect chondrocytes and the cartilage matrix. However, considering that HIV was reported to be able to infect chondrocytes [32], the more direct association between HIV/ART and cartilage matrix needs to be further explored.

Synovial inflammation can exacerbate cartilage degeneration by secreting more catabolic and pro-inflammatory mediators [33]. In turn, cartilage breakdown products also promote the inflammation of synovium [33, 34]. There is evidence that synovitis can be present at all stages of knee OA and is associated with cartilage destruction, knee pain and the progression of knee OA [21, 33, 34]. IPFP signal alteration and knee joint effusion have been reported to be surrogate markers for synovitis on non-contrast MRI, which were referred to as “Hoffa-synovitis” and “effusion-synovitis” respectively [20, 21]. As a local adipose tissue, abnormal IPFP and SPFP can secrete proinflammatory cytokines and adipokines, and thus may aggravate the local synovial inflammation [22, 35]. Studies have described the MRI features of OA-associated Hoffa-synovitis, which presents with distinguishable non-diffuse, heterogeneous alteration in signal intensity, often involving characteristically discrete portions of the IPFP and/or surrounding structures [17, 20–22]. In our study, two out of ten HIV-infected subjects presented a diffuse and homogeneous increased signal intensity throughout the whole IPFP, which was different from the imaging features of OA-associated Hoffa-synovitis and was not observed in the 20 HIV negative subjects. This special Hoffa-synovitis has been confirmed to be a non-specific inflammation, primarily composed of synovial proliferation and chronic nonspecific perivascular inflammatory cell infiltration [18]. The histological changes of SPFP signal alteration in HIV positive individuals remains unclear. However, in previous knee OA studies have been reported that SPFP signal changes may be similar to that observed in the IPFP which is characterized by inflammation, swelling, hypertrophy and fibrosis [23, 24]. Moreover, we found more severe effusion-synovitis at all timepoints in PLWH compared to controls. This difference likely results from the chronic inflammatory status promoting the release of proinflammatory mediators and could explain why HIV-infected subjects had more severe Hoffa-synovitis and SPFP synovitis at baseline.

Unexpectedly, the HIV group had a lower subchondral cysts scores compared to HIV-negative controls. Currently, two theories have been proposed to explain the origin of subchondral cysts in knee OA. The “synovial fluid intrusion theory” [36] posits that joint fluid intrudes into the subchondral bone via the breached subchondral plate, leading to the formation of cystic cavities. However, the “bony contusion theory” [37] suggests that cysts are the result of traumatic bone necrosis after impact of two opposing articular surfaces. Since HIV viral proteins have previously been reported to enhance osteoclast activity and inhibit osteoblast activity and certain ART combinations are known to detrimentally affect bone structure [38–40], our findings of lower subchondral cyst scores in PLWH seem counter-intuitive. However, given the complex effects of HIV and ART on bone resorption and bone formation, additional mechanisms may be involved reducing the development of subchondral cysts.

Our study has several limitations. First, the sample size was small; however, this is, to the best of our knowledge, the largest longitudinal study that has been performed to date in subjects with HIV. Studies have reported gender differences in knee OA and HIV infection [41–43]. However, due to the small sample size, we did not analyze the gender differences on cartilage degeneration and knee structural changes in HIV-infected subjects, which needs to be explored in future studies with larger sample sizes. Also, we evaluated the structural abnormalities using the WORMS scoring system which contributed to a more detailed assessment of the knee structural changes, but this system may not be sensitive enough to monitor more subtle changes of cartilage composition. Quantitative measurements can help us to obtain more accurate and compositional information which is necessary to further clarify the association between treated HIV infection and the progression of knee OA and will be our future research direction. Moreover, in this study we did not investigate the relationship between serum and synovial fluid biomarkers and their relation to HIV and OA. Synovial fluid biospecimens were not available in the OAI database. We previously investigated the relationship between serum and imaging biomarkers in a larger cohort and only found weak relationships [44]. Because of the small number of subjects we did not analyze these relationships in this study. Larger scale studies are required to investigate these biomarkers in subjects with HIV and their relation to OA in the future. Despite these limitations, we believe this study is valuable and provides novel information on the effects of HIV of knee joint degeneration.

## Conclusions

Our study showed a more heterogeneous and disordered cartilage matrix composition in PLWH on ART, suggesting that treated HIV infection may accelerate the degeneration of the knee cartilage matrix. In addition, compared with the HIV negative group, PLWH had significantly more severe knee joint effusion over 8 years. Moreover, at baseline PLWH had significantly more signal abnormalities of the IPFP and the SPFP, consistent with more severe joint effusion and knee synovitis. Our results suggest that treated HIV infection is associated with compositional changes of the cartilage matrix and increased knee joint inflammation, but findings appear not to have a significant impact on structural knee degeneration.

## Additional files

Additional file 1: Specific OAI datasets used in this study. (DOCX 13 kb)

Additional file 2: Medication regimens for participants with treated HIV infection. (DOCX 15 kb)

Additional file 3: The number of participants with available images at each point in time. (DOCX 15 kb)

## Abbreviations

ART: Antiretroviral therapy
BMEP: Bone marrow edema pattern
BMI: Body mass index
CI: Confidence interval
CV: Coefficients of variation
DESS: Dual-echo steady-state
GLCM: Gray level co-occurrence matrix
ICCs: The intra-class correlation coefficients
IPFP: Infrapatellar fat pad
IW: Intermediate-weighted
K&L: Kellgren-Lawrence
MetS: Metabolic syndrome
MSME: Multislice multiecho
NIH: National Institutes of Health
OA: Osteoarthritis
OAI: Osteoarthritis Initiative
PLWH: People living with HIV
SE: Spin-echo
SI: Signal intensity
SPFP: Suprapatellar fat pad
TE: Echo time
TR: Repetition time
TSE: Turbo spin-echo
WORMS: The whole-organ magnetic resonance imaging score
