# Supplementary material for: Is treated HIV infection associated with knee cartilage degeneration and structural changes? A longitudinal study using data from the osteoarthritis initiative
Source: BMC Musculoskelet Disord. 2019 May 4;20:190. doi: 10.1186/s12891-019-2573-5 (PMC6500016; doi:10.1186/s12891-019-2573-5)
Supplement: Supplementary file 1 — Specific OAI datasets used in this study. (DOCX 13 kb) [file 12891_2019_2573_MOESM1_ESM.docx]

**Specific OAI datasets used in this study:**

Specific OAI datasets used were clinical datasets 0.2.3, 1.2.2, 3.2.1, 10.2.2; medication inventory datasets 0.2.2, 1.2.1, 3.2.1, 5.2.1, 6.2.1, 8.2.1, 10.2.1; central radiograph reading datasets kXR SQ 0.8, 1.8, 3.7, 5.7, 6.5, 8.2, 10.2; physical exam and measurements datasets 0.2.2, 3.2.1, 6.2.1, 8.2.1, 10.2.1; and imaging datasets 0.E.2, 1.E.2, 3.E.2, 5.E.2, 6.E.2, 8.E.2, 10.E.2.
