# Supplementary material for: Is treated HIV infection associated with knee cartilage degeneration and structural changes? A longitudinal study using data from the osteoarthritis initiative
Source: BMC Musculoskelet Disord. 2019 May 4;20:190. doi: 10.1186/s12891-019-2573-5 (PMC6500016; doi:10.1186/s12891-019-2573-5)
Supplement: Supplementary file 2 — Medication regimens for participants with treated HIV infection. (DOCX 15 kb) [file 12891_2019_2573_MOESM2_ESM.docx]

**Medication regimens for participants with treated HIV infection.**

| Participants with treated  HIV infection (n=10) | Medication name**^a^** | Duration of medication use |
| --- | --- | --- |
| 1 | Intelence, Isentress, Norvir, Prezista, Truvada | > 1 year |
| 2 | Kaletra, Truvada | > 3 years |
| 3 | Epivir, Norvir, Viread | > 3 years |
| 4 | Combivir, Sustiva | > 5 years |
| 5 | Atripla | > 1 year |
| 6 | Epzicom, Kaletra, Reyataz | > 3 years |
| 7 | Kaletra, Lexiva, Truvada | > 5 years |
| 8 | Norvir, Reyataz, Trizivir | > 1 year |
| 9 | Atripla | > 1 year |
| 10 | Atripla | > 5 years |

**a:** The ingredients of each medication are listed in brackets: Intelence (Etravirine), Isentress (Raltegravir), Norvir (Ritonavir), Prezista (Darunavir), Truvada (Tenofovir, Emtricitabine), Kaletra (Ritonavir, Lopinavir), Epivir (Lamivudine), Viread (Tenofovir), Combivir (Lamivudine, Zidovudine), Sustiva (Efavirenz), Atripla (Efavirenz, Emtricitabine, Tenofovir), Epzicom (Abacavir, Lamivudine), Reyataz (Atazanavir), Lexiva (Fosamprenavir), Trizivir (Abacavir, Lamivudine, Zidovudine).
