# Supplementary material for: Is treated HIV infection associated with knee cartilage degeneration and structural changes? A longitudinal study using data from the osteoarthritis initiative
Source: BMC Musculoskelet Disord. 2019 May 4;20:190. doi: 10.1186/s12891-019-2573-5 (PMC6500016; doi:10.1186/s12891-019-2573-5)
Supplement: Supplementary file 3 — The number of participants with available images at each point in time. (DOCX 15 kb) [file 12891_2019_2573_MOESM3_ESM.docx]

**The number of participants with available images at each point in time.**

| Participants (n=30) | Side^a^ | Baseline | 12 month | 24 month | 36 month | 48 month | 60 month | 72 month | 96 month |
| --- | --- | --- | --- | --- | --- | --- | --- | --- | --- |
| HIV group (n=10) | R | 10 | 7 | 7 | 6 | 5 | 1 | 5 | 4 |
|  | L | 10 | 1 | 6 | 4 | 5 | 1 | 5 | 4 |
| Control (n=20) | R | 20 | 14 | 15 | 16 | 14 | 0 | 14 | 14 |
|  | L | 20 | 14 | 15 | 10 | 14 | 0 | 14 | 14 |

**a:** The number of participants with available images of left and right knees at each timepoint are provided separately.
